# Recent Insight on Regulations of FBXW7 and Its Role in Immunotherapy

**DOI:** 10.3389/fonc.2022.925041

**Published:** 2022-06-24

**Authors:** Liangliang Xing, Leidi Xu, Yong Zhang, Yinggang Che, Min Wang, Yongxiang Shao, Dan Qiu, Honglian Yu, Feng Zhao, Jian Zhang

**Affiliations:** ^1^Department of Pulmonary Medicine, Xijing Hospital, Air Force Medical University, Xi’an, China; ^2^Department of Anus and Intestine Surgery, The 942th Hospital of Joint Logistics Support Force, Yinchuan, China; ^3^Department of Hemato-Oncology, The 942th Hospital of Joint Logistics Support Force, Yinchuan, China

**Keywords:** FBXW7, ubiquitination, epigenetic regulation, immunity, immunotherapy

## Abstract

SCF^FBXW7^ E3 ubiquitin ligase complex is a crucial enzyme of the ubiquitin proteasome system that participates in variant activities of cell process, and its component FBXW7 (F-box and WD repeat domain–containing 7) is responsible for recognizing and binding to substrates. The expression of FBXW7 is controlled by multiple pathways at different levels. FBXW7 facilitates the maturity and function maintenance of immune cells *via* functioning as a mediator of ubiquitination-dependent degradation of substrate proteins. FBXW7 deficiency or mutation results in the growth disturbance and dysfunction of immune cell, leads to the resistance against immunotherapy, and participates in multiple illnesses. It is likely that FBXW7 coordinating with its regulators and substrates could offer potential targets to improve the sensitivity and effects of immunotherapy. Here, we review the mechanisms of the regulation on FBXW7 and its tumor suppression role in immune filed among various diseases (mostly cancers) to explore novel immune targets and treatments.

## 1. Introduction

Degradation is one of most significant bioprocesses of metabolism in almost all forms of life. For most eukaryotic cells, three pathways were discovered to degrade multiple proteins: (1) lysosomal pathway, (2) caspase pathway, and (3) ubiquitin proteasome pathway. Among them, the ubiquitin-proteasome pathway is the most irreplaceable specific protein degradation pathway, which can participate in various biological processes including cell proliferation, division, differentiation, and apoptosis, by promoting protein degradation (1, 2). The ubiquitin proteasome system (UPS) performs its protein-degrading function by three enzymes: the ubiquitin-activating enzyme (E1), the ubiquitin-conjugating enzyme (E2), and the ubiquitin ligase (E3). Among them, E3 ubiquitin ligase is the most critical component of UPS that can specifically recognize proteins to complete their ubiquitination. Emerging evidence exhibits that E3 shows a tendency weighing more in tumor suppressing than that in activating (3, 4). Skp1–Cullin1–F-box (SCF)^FBXW7^ consisting of Skp1, CUL1, F-box, and RBX1, is a well-learned category of E3 ligase in Really Interesting New Gene (RING) family (5). FBXW7 (F-box and WD repeat domain–containing 7) is usually deemed as a negative regulator of human cancers and is the most crucial F-box protein in E3 ligase so far (6).

Studies have indicated that FBXW7 is also involved in the regulation of immunity (7–11). Theories have explained the correlation between immunity and tumorigenesis, including tumor immunosurveillance and tumor immunoediting theory (12). Tumor immunoediting theory is a refinement of tumor immunosurveillance to explain the immune evsion of mutated cells and the progression of tumors. According to this theory, tumors develop in three stages. The first step is the elimination phase—the healthy body detects and eliminates mutated cells through immune surveillance; the second is the equilibrum phase—the immune system is so vulnerable that is uncapable of clearing out all of the mutated cells and leaves a significant number of the tumor cells remaining inside the body; and the final step is the escape phase—the tumor cells take a dominant position and become resistant to the attack launched by the immune system with the emergence of clinical signs and symptoms. Because immunity shows a close correlation with tumorigenesis, immunotherapy has been explored to fight against multiple cancers (13). Multiple immunotherapies have been developed, such as tumor vaccine, Bacille Calmette-Guerin (BCG), chimeric antigen receptor–engineered T lymphocytes, adoptive cellular therapy, and immune checkpoint inhibitors (ICIs). Among them, ICIs (including anti–PD-1/anti–PD-L1 and anti–CTLA-4) are mostly applied into the clinic, especially anti–PD-1/anti–PD-L1. Three immune patterns were characterized according to the anti-tumor reaction to PD-L1/PD-1 treatment (14). The first is the immune-inflamed phenotype featured by the existence of CD4- and CD8-positive T cells in the tumor parenchyma. The second is the immune-excluded phenotype featured by plentiful immune cells existing in the stroma without the ability of invading tumor parenchyma. The last one is the immune-desert phenotype featured by the absence of T cells in either parenchyma or stroma. A lot of research studies have found that SCF^FBXW7^ acting as a tumor suppressor is involved in tumorigenesis and resistance to immunotherapy by maintaining immune evasion (9, 15, 16).

This review mainly focuses on the FBXW7 functions in different cancers immunotherapy based on its structure and regulations in different levels, its involvement in multiple biological processes, as well its effect on immune cells and cytokines, which eventually draws a blueprint of targeting FBXW7 in immunotherapy.

## 2. Skp1–Cullin1–F-Box Protein Complex

The UPS plays a significant role in multiple bioprocess including cell propagation, division, and differentiation to decide the destiny of cell (7). It consists of three functionally interconnected enzymes: E1, E2, and E3. RING finger–type proteins and Homologous to the E6-AP Carboxyl Terminus (HECT) domain–type proteins are the largest two families of E3 ligase. In the RING family, E3 ligases transfer ubiquitin molecules directly from the E2 ubiquitin complex to the substrate without binding to ubiquitin molecules. The SCF ubiquitin ligase complex is a crucial member mainly composed of four units: Skp1 (composed of 163 residues), CUL1 (composed of 776 residues), F-box (composed of ~430 to >1,000 residues), and RBX1 (also called ROC1, composed of 108 residues) (17–20). CUL1 functions as a skeleton protein to interplay with the remaining three subunits (20). CUL1 interacts with RBX1via C terminus while it interacts with SKP1 *via* its N terminus. Moreover, F-box interacts with Skp1 *via* the F-box motif (21). SCF complex is deemed as one of the critical controllers of the mechanism in cell cycle for they regulate pivotal proteins progressing cell cycle (22). The F-box is the part of SCF complex responsible for recognizing and binding substrates. So far, nearly 70 F-box proteins have been found in humans as well as in other species (23). These proteins are further divided into three categories: Those rich in leucine repeats are called FBXL; the domain containing WD40 is called FBXW; the others are FBXO (with another or without motif except F-box protein) (24). FBXW7 (F-box with 7 tandem WD40 repeats) is the most famous FBXW protein for its significance in cellular processes including cell proliferation, division, and differentiation and is also known as FBW7, hAgo, hCDC4, and Sel10 (7).

## 3. FBXW7—The Most Critical F-Box Protein in the RING Family of E3 Ubiquitin Ligase

### 3.1. Structure and Locations

FBXW7 is the most widely researched F-box protein for its tumor suppression role up to now. It was first identified in budding yeast in 1973 by Hartwell et al. and then named CDC4 (25). This gene is highly conserved in multiple species. During the research on regulation of SEL-10 to LIN-12, the conserved gene CDC4 was also found in human cells and was named FBXW7 in terms of its structure, attracting increasing attention from then (26–28). FBXW7 gene that consists of 13 coding exons and 4 non-coding exons is located in the 4q31q.3 region of the human chromosome (a region frequently associated with deletion mutations in human tumors), and the gene length of FBXW7 is approximately 210 kb (6, 29). According to the difference between the 5′ untranslated region (5’UTR) and N-terminal coding region, the spliced variants of FBXW7 are divided into three subtypes: FBXW7α, FBXW7β, and FBXW7γ. Same gene as it comes from, the corresponding proteins translated by the three subtypes are located differently in subcellular regions: FBXW7α is located in the cytoplasm; FBXW7β is located in the cytoplasm; FBXW7γ is located in the nucleolus (30a). Different localization of FBXW7 can regulate their respective functions, which may be related to different pathways to bind to substrates. The three subtypes of FBXW7 share the following important conserved sequences: (1) the F-box domain, performing the function of recruiting SCF complexes through Skp1; (2) D domain, promoting the formation of FBXW7 dimer; and (3) WD40 domain, responsible for substrate recognition. Apart from the distinguishment in intracellular localization, three subtypes of FBXW7 also expressed discrepantly in different tissue types. A study in 2002 found that FBXW7α is widely expressed in human tissues, whereas FBXW7β and FBXW7γ are highly expressed mainly in the heart, brain, and skeletal muscle (29). Four years later, another mice model study came to similar conclusions (31). Currently, research studies lay emphasis mainly on the function of FBXW7α, whereas the other two subtypes of FBXW7-related biological studies are relatively rare.

### 3.2. Regulation of FBXW7

#### 3.2.1. FBXW7 Regulation at Transcriptional Level

Since we have known the structure and locations of FBXW7, we want to figure out the mechanisms on how it is regulated. Regulation of FBXW7 at transcriptional level represents a tendency to negatively regulating the expression of FBXW7. RAN-binding protein 10 (RANBP10) promotes the stabilization of c-myc *via* binding to the region P4 of FBW7 promotor and inhibiting its transcription, which induces the progression of glioblastoma (32). A complex of PHF1/PRMT5–WDR77/CRL4B represses transcription of FBWX7 by taking up its promotor, which leads to the progression of cancer (33). C/EBPδ functions as an inhibitor binding to the promoter of FBW7α and decreases the abundance of FBXW7α mRNA, contributing to improved activity of HIF-1 *via* stabilizing mTOR (34). Furthermore, a feedback loop consisting of FBXW7, Hes5, and NOTCH intracellular domain (NICD) is involved in the inactivation of FBXW7 mRNA (35). In this loop, Hes5 binds to the N-box in the promotor of FBXW7 to suppress its transcription, which can affect the fate of intestinal and neural stem cells. Moreover, the inactivation of FBXW7 mediated by Hes5 participates in the inhibition of TGF-β pathway as well (36). TRIP13 confers the carcinogenicity of glioblastoma *via* binding to the promoter near FBXW7 gene and further stabilizing c-myc by suppressing expression of FBXW7 (37). Intriguingly, P53 activates the transcription of FBXW7β by binding to a site in exon 1b, acting as a resistance against genotoxic pressure from UV radiation and adriamycin (38).

#### 3.2.2. Epigenetic Regulation of FBXW7

##### 3.2.2.1. Methylation and Demethylation Modification

Several studies have uncovered the correlation of DNA methylation with FBXW7 expression. In the study of Akhoondi et al., they found that the ratio of methylation of the FBXW7β promoter in cancer cell line is 43%, wherereas the number in primary breast cancer is 51% (39). Data suggested that FBXW7β of methylated group was downregulated both in cancer cell line and in primary breast cancer compared with the unmethylated group. They also found that although methylation was connected with advanced tumors, the hazard ratio (HR) for patients’ death with FBXW7β methylation on the opposite showed a downtrend. Gu et al. discovered that, with the methylation of the CpG sequence in FBXW7β’s promoter, a significant decline of the expression of FBXW7β was observed (40a). The methylation in FBXW7β promoter was found to be positively associated with thymoma histological subtype that presents a positive correlation with prognosis of patients (41b). In addition, the methylation level of FBXW7 gene 5′ upstream areas of p53-mutated samples was significantly higher than that of wild-type samples, which may due to the suppression of FBXW7 expression by p53 mutations through the hypermethylation in designated areas (42). More epigenetic silencing of FBXW7 was exhibited in human papillomavirus-IMM (HPV-IMM) than HPV16-KRT, which may work in the stratification of cervical squamous cell carcinoma (CSCC) affected by HPV16 to provide reasonable treatment for patients (43). Furthermore, lysine demethylase 5B (KDM5B), a histone demethylase of FBXW7, was unveiled to suppress FBXW7 expression *via* demethylation of H3K4me3 at promoter region (44). The mechanism of FBXW7 epigenetic modulation has been applied into clinical treatment for patient of lung cancer as decitabine is able to demethylate the epigenetically silenced FBXW7 gene and reactivate it (45)

##### 3.2.2.2. Histone Acetylation

In addition to DNA methylation, histone acetylation is also implicated in regulation of FBXW7. A research to detect the DNA methylation, histone methylation, histone acetylation, and chromatin remodeling uncovered that H3K27 acetylation suppressed by the mutation or knockdown in CREBBP or EP300 in B-lymphoma cells weakened the expression of FBXW7, leading to the activation of NOTCH pathway and thereby caused the tumor-associated macrophage (TAM) polarizing to M2 phenotype and proliferation of tumor cells (46). Histone acetylation could also work synergistically with DNA methylation. Data acquired from The Cancer Genome Atlas (TCGA) revealed that high DNA methylation of the FBXW7 was accompanied with high KDM5c (a histone demethylase) expression, which might ascribe to the recruitment of DNMT3b induced by interaction of KDM5c and H3K4me3 of FBXW7 downstream so that the CpG of FBXW7 could be methylated, followed with inhibited FBXW7 expression (47a).

##### 3.2.2.3. Chromatin Remodeling

Chromatin remodeling is involved in the mediation of FBXW7 as well. An experiment conducted by Masayuki Hagiwara disclosed that the expression of FBXW7 was restrained by the overexpression of MUC1-1C, which activates the components, including MBD3, MTA1, and CHD4, of the nucleosome remodeling and deacetylation (NuRD) complex to facilitate Interferon Gamma Receptor 1 (IFNGR1) expression (48, 49). Another study demonstrated that FBXW7 has an intimated relation with chromatin remodeling through whole-exome sequencing of 57 cancers (50). Although the direct relationship between FBXW7 and chromatin remodeling was not demonstrated, we speculate that FBXW7 expression could be inhibited by means of chromatin remodeling. However, more work remains to be done to explore the deeper mechanisms. In this way, the substrate of FBXW7, IFNGR1, showed a trend of increasing expression and further promoted the tumorigenesis and metastasis of cancer (51).

Collectively, epigenetic modification regulates FBXW7 in three ways: methylation and demethylation modification, Histone acetylation and Chromatin remodeling.

##### 3.2.2.4. RNA Epitranscriptomic Modification of FBXW7

A previous research conducted by our team revealed that N6-methyladenosine (m^6^A) is involved in the methylated modification of FBW7 mRNA (52). M^6^A refers to the methylation of N6-adenosine in eukaryotic mRNA controlled by writers—methyltransferases, erasers—demethylases, and readers—binding proteins, which is a universal modification of mRNA and affects various pathphysiological processes including tumorgensis (53, 54). METTL3 is one of the methyltransferases which methylates m^6^A of FBXW7 mRNA and facilitates its translational efficiency to repress lung adenocarcinoma (52). Interestingly, not only is FBXW7 regulated through m^6^A, but it also regulates the m^6^A of other mRNAs. FBXW7 targets YTHDF2 protein, the m6A reader of BMF mRNA, rescuing the YTHDF2-mediated inactivation of BMF mRNA and repressing the growth and progression of ovarian cancer (55).

#### 3.2.3. Regulation of FBXW7 Mediated by Non-Coding RNA

##### 3.2.3.1. MicroRNA Regulation of FBXW7

Non-coding RNA also functions in regulation of FBXW7. MicroRNA (miRNA) is a kind of non-coding RNA that can suppress the mRNA and intervene the subsequent protein synthesis *via* binding to the 3′UTR. Plenty of miRNAs have been uncovered to bind to the 3′UTR of FBXW7 and inhibit the protein translation. Overexpression of miR-223 plays roles in different situations by the counteraction of FBXW7. MiR-223 functions in gastric cancer for carcinostasis and drug resistance (56, 57), gives rise to physiologic cardiac hypertrophy (58), and protects CRC cells against apoptosis and promotes its proliferation as well (59). Moreover, a KLF5/mi-R27a/FBXW7 axis was reported to enhance migration and invasion of ccRCC when FBXW7 was reduced by mi-R27a (60). Apart from involvement in cancer, downregulation of FBXW7 by miR-322 demonstrates a possible curing method to protect myocardium from ischemia/reperfusion injury. Collectively, there are still other miRNAs, such as miR129 (61), miR-92a-3p (62), miR182 (63), miR-27a-3p (64, 65), miR-212/132 (66), miR-223-3p (67), miR-103a-3p (68), miR-25 (69), miR−25−3p (70), miR-144 (71), miR-101 (72), miR-188-5p (73), and miR-500a-3p (74), functioning discrepantly to mediate different cell phenotypes but *via* the identical mechanism of targeting FBXW7.

##### 3.2.3.2. Long Non-Coding RNA Regulation of FBXW7

Long non-coding RNAs (lncRNAs) are non-coding RNAs with more than 200 nucleotides in length and without the function of coding proteins (75). LncRNAs take part in various biological activities by interplaying with DNAs, RNAs, and proteins (76, 77), which means lncRNAs regulate FBXW7 at different levels directly or indirectly. A lncRNA-associated-feedback loop was explored by Pengfei Zhang et al. demostrated that lncRNA-MIF (c-myc inhibitory factor) functioning as a competing endogenous RNA (ceRNA) for miR586 weakened the suppression of miR-586 on FBXW7 (78). Therefore, the substrate of FBXW7, c-myc, was repressed subsequently as FBXW7 was upregulated by the lncRNA-MIF–associated feedback loop and the aerobic glycolysis and pro-oncogenicity triggered by c-myc was eliminated. As c-myc was repressed by FBXW7, its induction to lncRNA-MIF was attenuated, which in turn results in the reduction of lncRNA-MIF abundance. Moreover, lncRNAs of similar roles served as miRNA “sponges” include MALAT1 (79), CASC2 (80), TINCR (81), MT1JP (82), FER1L4 (83), MIR22HG (84), TTN-AS1 (85), LINC00173 (86), and PADNA (87). Other mechanisms of lncRNAs regulating the expression of FBXW7 are displayed as well. A research carried out by Lianzhi Wu et al. revealed the pathway that FBXW7 directly recruited by MALAT1 contributed to the degradation of CRY2 (88). LncRNA BDNF-AS was capable of recruiting WDR5 to contribute to CpG island methylation of FBXW7, by which FBXW7 was downregulated and the ubiquitination of its substrate VDAC3 was diminished (89a). Another study verified that lncRNA SEMA3B-AS1 integrates with HMGB1, a transcription factor of FBXW7, and then facilitates the expression of FBXW7, resulting in the enhanced ubiquitination-mediated degradation (90). In addition, lncRNA TUG1 facilitates the expression of FBXW7 at protein level instead of mRNA level to destabilize SIRT1, leading to the abrogation of neuronal mitophagy (91).

##### 3.2.3.3. Circular RNA Regulation of FBXW7

Circular RNA (circRNA) is distinguished by its structure of covalently closed loop without polyadenylated tail or 5′ to 3′ polarity (92). Parallel to lncRNAs, most circRNAs regulate FBXW7 as miRNA “sponges”. For instance, hsa_circ_11780 (93), circFBXW4 (94), Hsa_circ_001988 (95), circ_CLASP2 (96), circBRAF (97), circ_0000094 (98), hsa_circ_0001306 (99), hsa_circ_0022742 (100), circPSD3 (101), and circKL (102) were confirmed to sponge different miRNAs to impact the expression of FBXW7, respectively. Intriguingly, although the majority of circRNAs are deemed unable to code proteins as non-coding RNAs, a special circRNA associated with FBXW7 has been confirmed the function of coding protein. Circ-FBXW7 was a product of the circulation of cyclization of exons 3 and 4 in the FBXW7 gene that can code a neo-protein named FBXW7-185aa (103, 104). One study revealed that the FBXW7-185aa coded by circ-FBXW7 regulating FBXW7 protein *via* competitively binding to USP28 in glioma (104). Another study discovered two pathways in triple-negative breast cancer to control the expression of FBXW7: One displayed that circ-FBXW7 sponges miR-197-3p to promote the expression of FBXW7. The other demonstrated a similar pathway to that in glioma—FBXW7-185aa competitively binding to USP28 to protect the function of FBXW 7 (103).

In brief, three kinds of non-coding RNAs, including miRNAs, lncRNAs, and circRNAs, regulate the expression of FBXW7 in their own ways. In addition, some miRNAs work synergistically with lncRNAs or circRNAs to fulfill the function together.

#### 3.2.4 Dimerization of FBXW7

Although the protein translation process has been completed, the abundance of FBXW7 can also been influenced. Dimerization is not only a widely phenomenon for all F-box proteins but also a critical regulatory mechanism for FBW7-mediated ubiquitination to substrates as well (105–108). FBXW7 owns three shared domains among all different isoforms as depicted, and the D domain mainly mediates FBXW7′ dimerization (107). Dimerization of FBXW7 may exert the following effects: (1) impacting the location of different isoforms; (2) raising the possibility for several substrates to be regulated by FBXW7; (3) capable of regulating the autoubiquitination of FBXW7; (4) functioning as a buffer to endure mutations that impair the FBXW7 substrate and hinder the substrate degradation mediated by monomeric FBXW7; (5) and increasing ubiquitination rate and processivity (5, 105, 108). However, take the examples of c-myc and cyclin E (108), widely seen as it is, not all substrates ubiquitination need dimerization of FBXW7. Intriguingly, a latest research revealed that LSD1, often regarded as a demethylase of histone, directly bound to FBXW7 to disturb the formation of dimerization to facilitate autoubiquitination rather than activate the demethylation of FBXW7, which might offer a new target for cancer treatment (109).

#### 3.2.5 Phosphorylation of FBXW7

Phosphorylation of FBXW7 also occurs after FBXW7 translation. Activation of ERK1/2 kinase occurs in many drug-resistant tumor cells (110, 111), and FBXW7 is phosphorylated at Thr^205^ and further degraded by UPS (112). The instability of FBXW7 caused by phosphorylation has been verified in many experiments. Mun et al. investigated that Erk1/2 kinase participated in the inhibition of FBXW7 expression in drug-resistant cells (A549-Taxolr cells and T47D-Doxr cells), which led to an increase in heat-shock factor 1 (HSF1) and promoted transcriptional activation of MDR1 (113). This phosphorylation-dependent regulation of FBXW7 by ERK1/2 was also confirmed by other studies. Shu et al. found that ERK1/2-mediated phosphorylation of FBXW7 was involved in transcriptional regulation of FOS-like 1 (Fra-1) by Neuregulin 1 *via* stabilizing downstream c-myc that could bind to the promoter of Fra-1, thereby promoting metastasis of triple negative breast cancer (114). Another example of FBXW7 phosphorylation by ERK kinase at the T^205^ residue was verified likely to affect the ferroptosis of pancreatic carcinoma cells (115). In addition, FBXW7-myc-PLK1 forms a regulatory loop that controlled neuroblastoma tumor progression, in which FBXW7 was phosphorylated at Thr^284^ and Ser^58^ by PLK1 (116). Similar regulatory loop was also discovered in medulloblastoma (117). Phosphorylation of FBXW7 by related kinases not only contributes to the degradation of FBXW7 but also improves the catalytic activity of FBXW7 to downstream substrates. The process of FBXW7 phosphorylation at Ser^227^ mediated by serum and glucocorticoidregulated kinase 1 (SGK1) or Phosphoinositide 3-kinase (PI3K) enhances the ubiquitination ability of FBXW7 (118, 119). Furthermore, phosphorylation of FBXW7α at Ser^10^/Ser^18^ mediated by protein kinase (PK) C was found both *in vitro* and in mammals, and phosphorylation of Ser^10^ had been validated to affect nuclear localization of FBXW7α (120). Other results had been obtained both in human and Xenopus eggs in regard to PKC phosphorylation of FBXW7 that FBXW7α phosphorylated by PKC at Ser^18^ residues occurred during mitosis, which stabilized FBXW7 itself but interfered with ubiquitination of downstream cyclin E (121).

#### 3.2.6 Autoubiquitination of FBXW7

Not only can FBXW7 degrade its substrates in the ubiquitination dependent way, but it is also competent for its autoubiquitination. It is a universal phenomenon that F-box is ubiquitinated for its necessity to strike the functional balance of SCF complex *via* the autoubiquitination (122, 123). F-box is more apt to be degraded compared with other components in SCF complex for its instability. The process of F-box ubiquitination takes place within the SCF complex itself without the assistance of other F-box proteins as the ubiquitination is required to be complete (124). Ubiquitin binding to FBXW7 was reported to facilitate ubiquitination and degradation of FBXW7, for which was predominant in the competition against the substrates of the FBXW7 (125). Moreover, FBXW7 autoubiquitination is also promoted through its dimerization (105). Another investigation revealed that CSN6 increased neddylation of Cullin-1 to contribute to FBXW7 autoubiquitination as a K48 linkage (126). In contrast, PML promotes FBXW7 expression *via* inhibiting ubiquitination and degradation of FBXW7 in the K48-linked way to enhance antiviral immunoreaction against influenza virus (127).

#### 3.2.7 Biomechanical Regulation of FBXW7

What we have mentioned above are biological pathways to regulate FBXW7 expression. Interestingly, a biomechanical pathway that controls FBXW7 was unveiled recently (128). The investigation conducted by Haiyan Zhang suggested that mechanical overloading downregulated FBXW7 in chondrocyte of patients with osteoarthritis. The critical step that they found contributing to FBXW7 suppression occurred in the synthesis of mRNA. As a result of FBXW7 downregulation, MKK7–JNK pathway was activated and further facilitated the senility of chondrocyte. It offers us a promising curing target on treatment osteoarthritis and a fire-new viewpoint to explore the undiscovered pathways of the regulation of FBXW7.

### 3.3. The Pattern of FBXW7 Binding to Its Substrates

#### 3.3.1. CPDs of Substrates

Previous studies found that a large proportion of substrates of FBXW7 was unable to bind FBXW7 E3 ligase until the CDC4 phospho-degrons (CPDs) of substrates were phosphorylated by protein kinase (129–131). Optimal sequences refer to CPDs sequences interacting with FBXW7 that contain essential residues. Specifically, there should be at least one hydrophobic amino acid at the sites −5, −3, −2, and −1; it is usually threonine or serine that takes up site zero; the +1 and +2 sites are generally proline; the +4 site may be one of serine, threonine, glutamate, and aspartic acid (7). Only when the 0 and +4 amino acids are phosphorylated by kinases can the substrate be discerned by the WD40 domain and participate in the UPS pathway for degradation. Intriguingly, not all CPDs of substrates follow this rule. CPDs with unfavorable residues of some proteins partially different from the optimal sequence are called semi-optimal CPDs. However, a portion of substrates CPDs remains undiscovered (7). In addition, some substrates possess more than one CPD sequence. KLF5 was reported to be degraded by FBXW7 with any of three CPDs phosphorylated by GSK-3β, which result in its repressed bio-activity in cell propagation (129). FBXW7 dimerization is likely to play a role in its interaction with the CPDs of protein as it was surveyed that the dimer structure can interact with the two CPDs of cyclin E to enhance the binding ability of cyclin E and promote its ubiquitination degradation (105, 107).

#### 3.3.2. UPS-Dependent Degradation of Substrates

**Figure 1:**
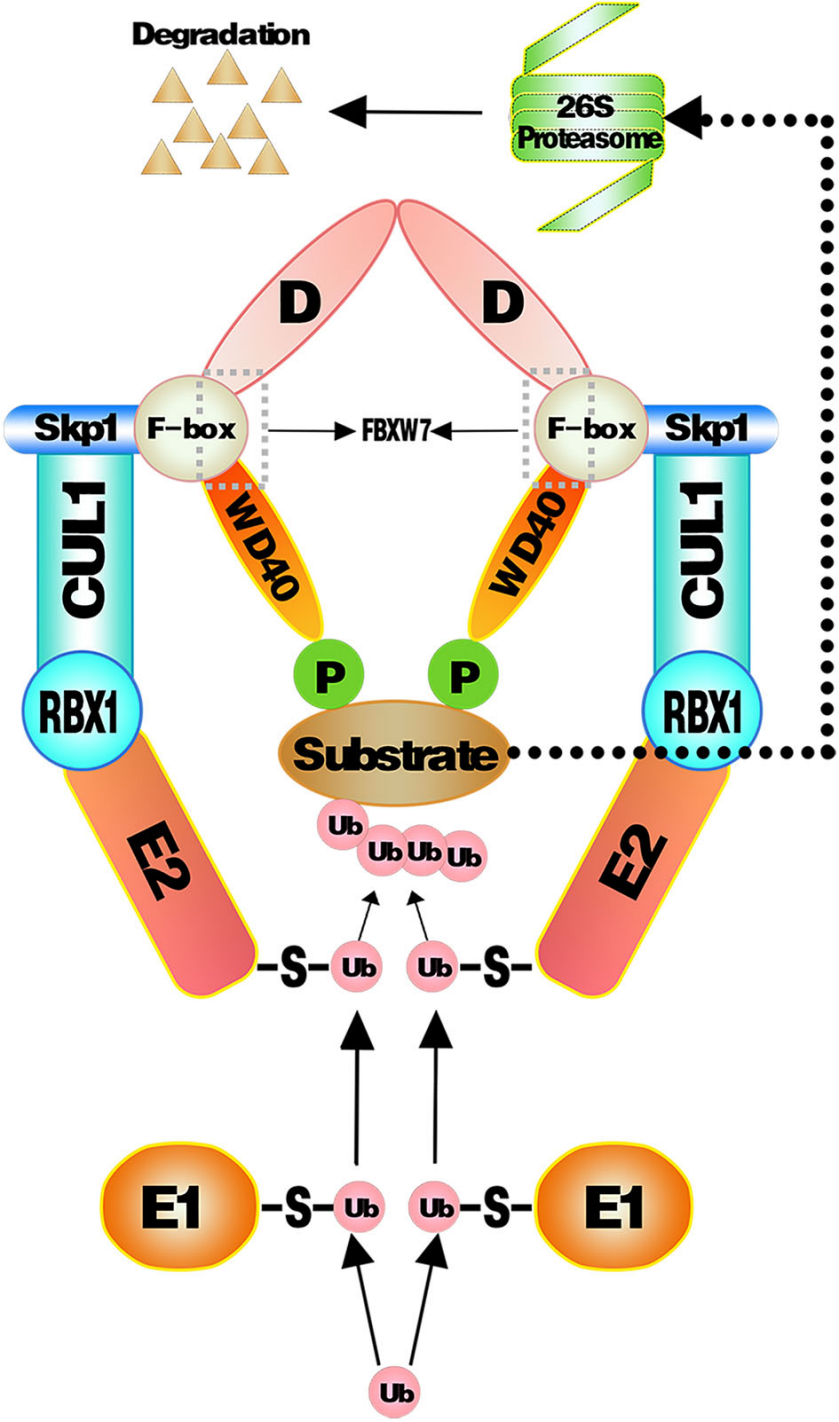
Substrates proteins targeted and ubiquitinated by SCF^FBXW7^ complex. Ubiquitin molecules are activated by E1 and then transferred to E2. E3 recognizes phosphorylated substrates and combined to E2 ubiquitin to transfer ubiquitin from E2 to substrates. As sufficient ubiquitin chains form onto substrates, they will be captured and cut into peptides in an ATP-dependent way by the 26S proteosome.

Multifarious substrates of FBXW7 have been previously reported before (7). E3 ligase recognizes the substrates once the CPDs of substrates are phosphorylated and then transfer ubiquitin molecules to the substrates (generally to a lysine side chain) to facilitate the ubiquitination of substrates (132). More than one poly-ubiquitin chain or multiple mono-ubiquitins are required to initiate the degradation (133). When substrates acquire enough ubiquitin chains, they will be captured and cut into peptides in an Adenosine Triphosphate (ATP)-dependent manner by the 26S proteosome (134). The UPS-dependent degradation of substrates mediated by SCF^FBXW7^ is shown in (**Figure 1**).

### 3.4. Interaction of Deubiquitinating Enzymes and FBXW7

Generally, as proteins are captured by 26S proteosome, deubiquitinating enzymes (DUBs) directly cut off ubiquitin from ubiquitin-binding proteins in various cell bioprocesses (135). Specifically, here, we introduce unusual pathways for DUBs regulating protein. USP28 (one of DUBs) could counteract the effect of FBXW7α degrading myc in the nucleus by forming a special complex with myc and FBW7α (136a; 137). Similarly, USP28 could stabilize HIF-1α by antagonizing FBXW7 rather than interacting with HIF-1α, which impacts the cancer cell activity and capillary formation (138). Analogous mechanism was observed on the regulation of NICD1 and c-jun in intestinal homeostasis and tumorigenesis (139). Interestingly, another study found that partial reduction of USP28 stabilizes FBXW7 and facilitates degradation of its substrates, whereas the absence of USP28 can facilitate FBXW7 ubiquitination, and overexpression of USP28 preferentially counteracts autocatalytic ubiquitination of FBXW7, leading to the increased stability of both FBXW7 and of its substrates simultaneously (140). USP36 is another DUB antagonizing the degradation of c-myc mediated by FBXW7γ to maintain the stability of c-myc in nucleolus (141–143). Moreover, USP9X interacts with FBXW7 and protects it from autoubiquitination, which results in the suppression on substrates of FBXW7 and maintain intestinal homeostasis *via* controlling c-myc and NOTCH1 (144). In short, DUBs not only interact with proteins to cut off ubiquitins but also interact with FBXW7 to influence its function on substrates.

## 4. FBXW7 in Immunity

### 4.1 FBXW7-Mediated Immune Evasion Promotes Carcinogenicity

Both the regulations and degradation functions of FBXW7 have been reviewed above, and we want to further explore whether it is involved in immunity for immunity has a crucial connection with most diseases including cancers. Immunity has an intimated correlation with the health of mammals from birth to death in controlling the equilibrium state of bodies and causing harmful response under unbalanced immune homeostasis. Chances are that immune evasion happens when immunity is suppressed. Studies have demonstrated that FBXW7 is involved in the regulation of immune evasion occurring both in anti-virus and anti-tumor immunoreaction. Porcine epidemic diarrhea virus (PEDV) was found to promote FBXW7 degradation in a UPS-dependent way to attenuate the antiviral reaction (145). Nonstructural protein (nsp2) encoded by PEDV was identified to be the component interplaying with FBXW7 and contributing to degradation of FBXW7 *via* disturbing the stability of retinoic acid-inducible gene I (RIG-I), which results in reduced production of IFN I (145, 146). The involvement of FBXW7 in immune evasion was also reported in cancer cells (15). Eyes absent homolog 2 (EYA2) could be recognized by SCF^FBXW7^ and further accept the final destiny of being degraded. FBXO7, one of the F-box proteins, is capable of binding and stabilizing EYA2 in an SCF-independent way to protect EYA2 against FBXW7-mediated degradation so as to facilitate AXL-mediated immune evasion (15).

As it is known, immune evasion is one of the mechanisms of tumorigenesis. Therefore, FBXW7 might be involved in immunity-related tumorigenesis and immunotherapy resistance.

### 4.2 FBXW7 Influences the Cancer Progression Through Its Effect on Immune Cells

#### 4.2.1. Macrophages

**Figure 2:**
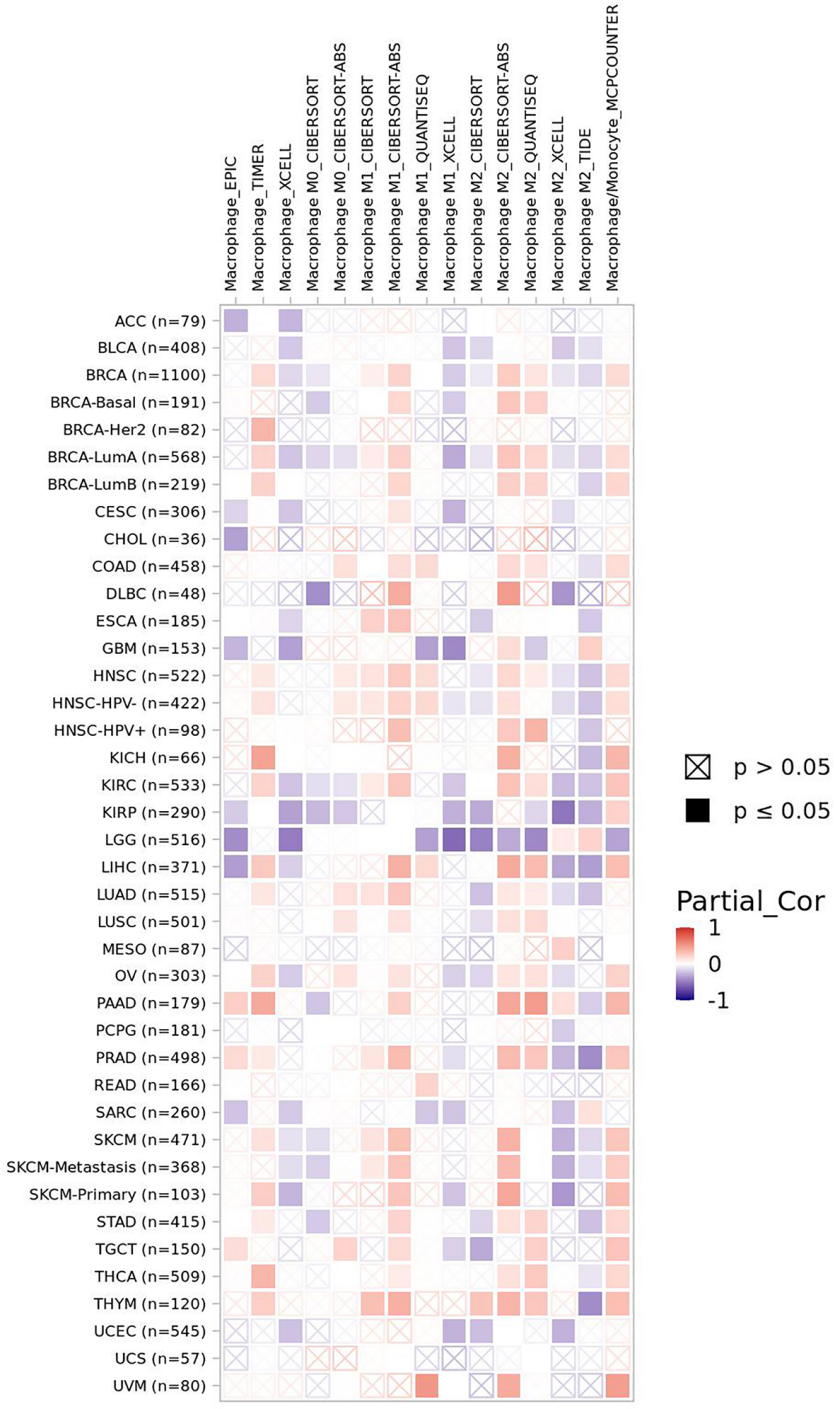
The correlation of FBXW7 expression with macrophages infiltration level in diverse cancer types (data from timer 2.0).

Macrophages are involved both in innate immune response and adaptive immune response. FBXW7 participates in the polarization of tumor-associated macrophages *via* different pathways (9, 46). The CCAAT/enhancer-binding protein-δ (C/EBPδ, Cebpd), an inflammation related gene regulated by NF-κB and ATF3, functions to amplify innate immune response *via* identifying the status of Toll-like receptor 4 (TLR4)–induced signals (TLR4 could induce the activation of macrophages) (16). FBXW7 was revealed to weaken the inflammatory pathway by targeting C/EBPδ as it was phosphorylated to further negatively regulate TLR4 and its reaction to ligand lipopolysaccharide (LPS) (147). Another research found that MiR-223 downregulated FBXW7 and TLR4 expression in macrophages, which modulated the inflammatory reaction of macrophage to LPS (148). However, when FBXW7 was repressed by estrogen receptor α (Erα) in breast cancer, high expression of C/EBPδ was revealed to attenuate the carcinogenicity of cancer cells through suppressing expression of the SNAI2, which is different from a previous research correlated with the role of C/EBPδ in breast cancer (34, 149). Mice lacking in FBXW7 show improved expression of chemokine C-C Motif Chemokine Ligand 2 (CCL2) in serum, which contributed to the recruitment of macrophages and monocytic myeloid-derived cells and then led to the metastasis of tumor (11). The similar negative relationship between FBXW7 and CCL2 was observed in serum of human later (150). By contrast, the deficiency of FBXW7 in CX3CR1hi macrophages enhanced the abundance of CCL2/CCL7 to induce intestinal inflammation (151). In the study of Zhang et al., FBXW7 inhibited by calcium/calmodulin-dependent protein kinase IV (CaMKIV) promoted the upregulation of mTOR in macrophages, resulting in the LPS-induced autophagy of macrophages subsequently (152). FBXW7 deficiency in myeloid cells promotes the recruitment of monocyte-macrophages in pulmonary tissue, facilitating the collagen deposition induced by bleomycin and finally developing into progressive pulmonary fibrosis (153). Moreover, the suppression of MCL-1 by FBXW7 in M2 macrophages demonstrated an improved apoptosis and repressed EMT phenotype of colon cancer cells (8). Data from timer 2.0 show that the expression of FBXW7 displays a positive correlation with macrophages infiltration level in colon adenocarcinoma (COAD) (**Figure 2**). A metabolism-related pathway of FBXW7 deficiency in myeloid results in the loss of substrate flux *via* pentose phosphate pathway, which causes a decreasing production of equivalents [nicotinamide adenine dinucleotide phosphate (NADPH) and Glutathione (GSH)] and then increases the reactive oxygen species in macrophages to promote the inflammation (154).

#### 4.2.2. NK Cells

**Figure 3:**
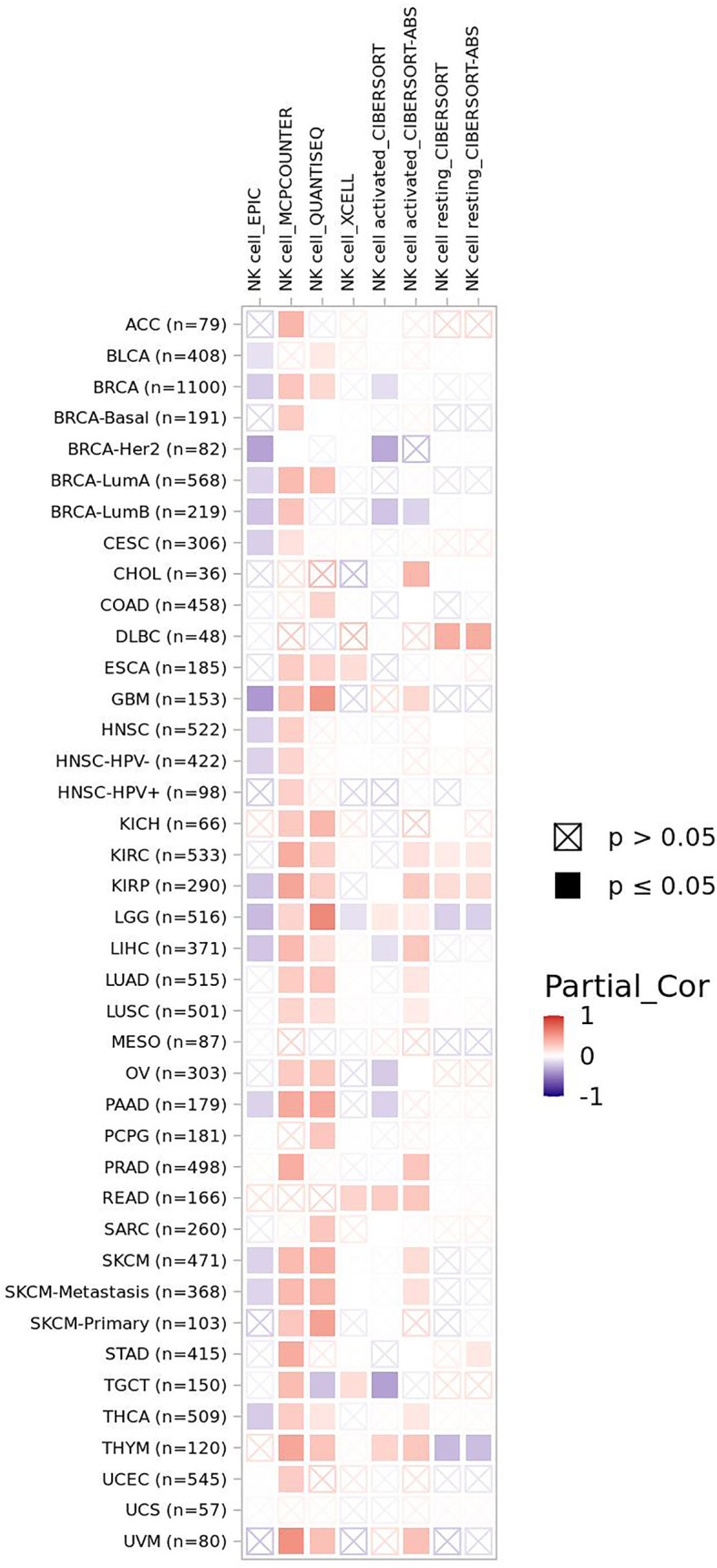
The correlation of FBXW7 expression with NK cells infiltration level in diverse cancer types (data from timer 2.0).

EYA2 mentioned above can be degraded by SCF^FBXW7^-mediated ubiquitination. Downregulation of EYA2 leads to weakened mesenchymal phenotypes, improved immunogenicity of cancer cells, decreased carcinogenicity including tumor growth and metastasis, increased infiltration level of natural killer cells (NK cells), and cytotoxic T cells. As a result, a favorable anti–PD-L1 therapy occurs in mice tumor models (15). The correlation between FBXW7 and NK cells demonstrates a positive interaction for curing tumors. Infiltration level of NK cells basically is positively associated with FBXW7 expression in different cancer (**Figure 3**).

#### 4.2.3. Lymphocytes

FBXW7 is a critical regulator to maintain the quiescence and self-renewal of hematopoietic stem cells (HSCs) *via* regulating four critical genes, including Ccnd1, Evi1, Pbx3, and Meis1, participating in differentiation of HSCs. On regulation of lymphocytes, FBXW7 deficiency of progenitors in bone marrow lose the ability to colonize the thymus, resulting in a significant shortage of T lymphocyte progenitors and an obvious decline of all B lymphocytes, and FBXW7 is considered as a driver gene for CLL ascribe to its mutations and degradation of NOTCH1 (155, 156).

##### 4.2.3.1. B Lymphocytes

**Figure 4:**
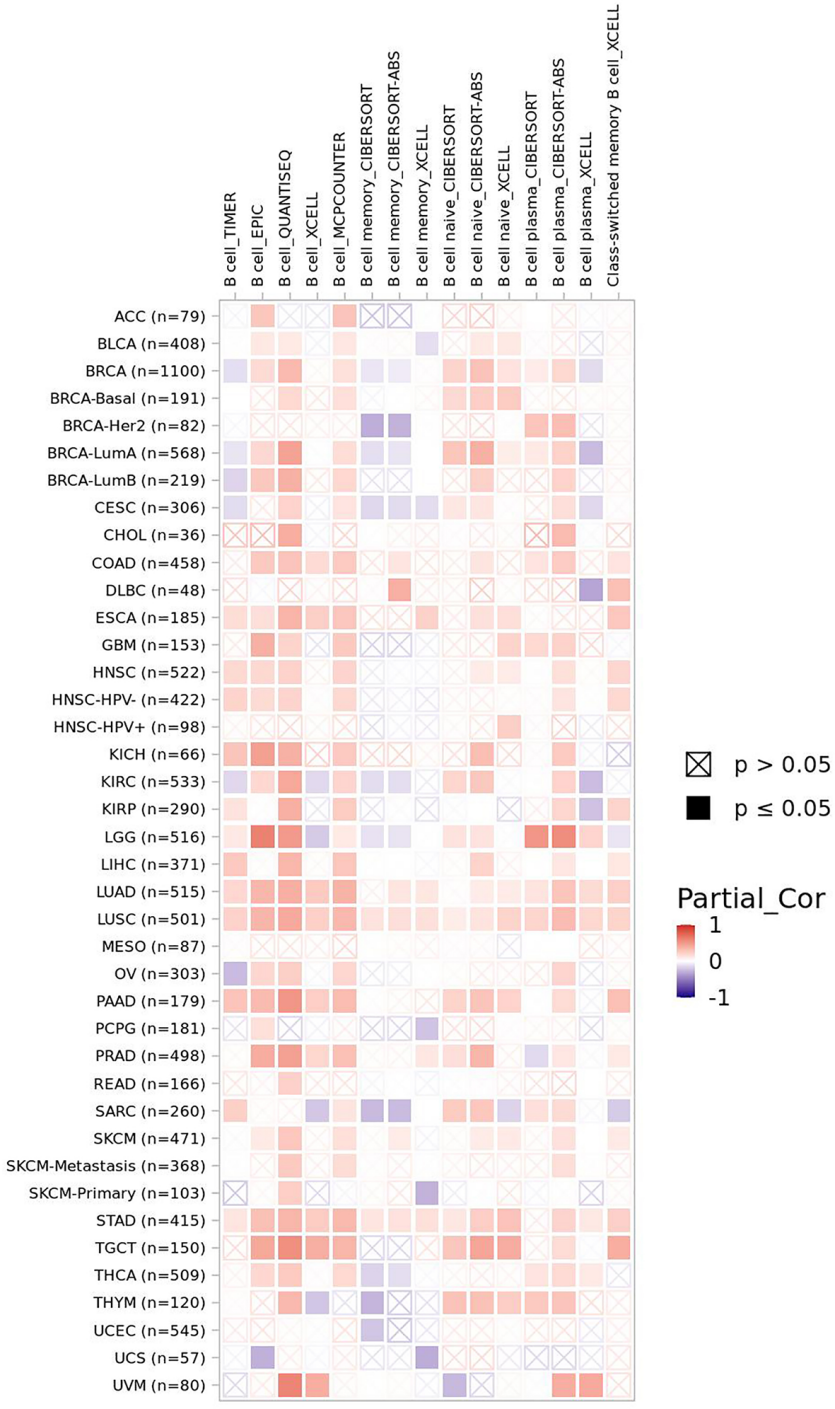
The correlation of FBXW7 expression with B-cell infiltration level in diverse cancer types (data from timer 2.0).

B lymphocyte plays an important role in keeping of immunity and immune tolerance (157). FBXW7 is of critical significance to maintain the mature B lymphocytes populations in mice. B-cell receptors (BCRs) stimulation of B cells lead to their apoptosis and stasis of proliferation and growth, owing to FBXW7 deficiency in mice (10). The volume of FBXW7-deficeient B cells is smaller that of normal group after accepting the stimulation of anti-IgM, displaying that FBXW7 matters a lot to BCR-mediated proliferation and survival of B cells (157). On condition that FBXW7 is absent in B cells, Ig class-switching is destroyed, functions of GC (germinal center) including CSR (class switch recombination) and affinity maturation of antibody are badly impaired, and memory antibody response is whittled, which might be the result of FBXW7 affecting BCL6, a protein of great importance to initiate and maintain the GC reaction (157). As shown in the mice model case of collagen-induced arthritis, a slower disease induction stage, a later disease onset, a lighter disease severity, a lower disease incidence, and a gentler joint destruction occur in FBXW7-deleted mice compared with control group, in accordance with the parallelly decreased anti-CII autoantibodies (157). Collectively, a latent treatment related to FBXW7 to cure GC-connected and autoantibody-induced autoimmune diseases is offered. FBXW7 expression presents a positive correlation of B-cell infiltration level in most cancers (**Figure 4**).

##### 4.2.3.2. T Lymphocytes

**Figure 5:**
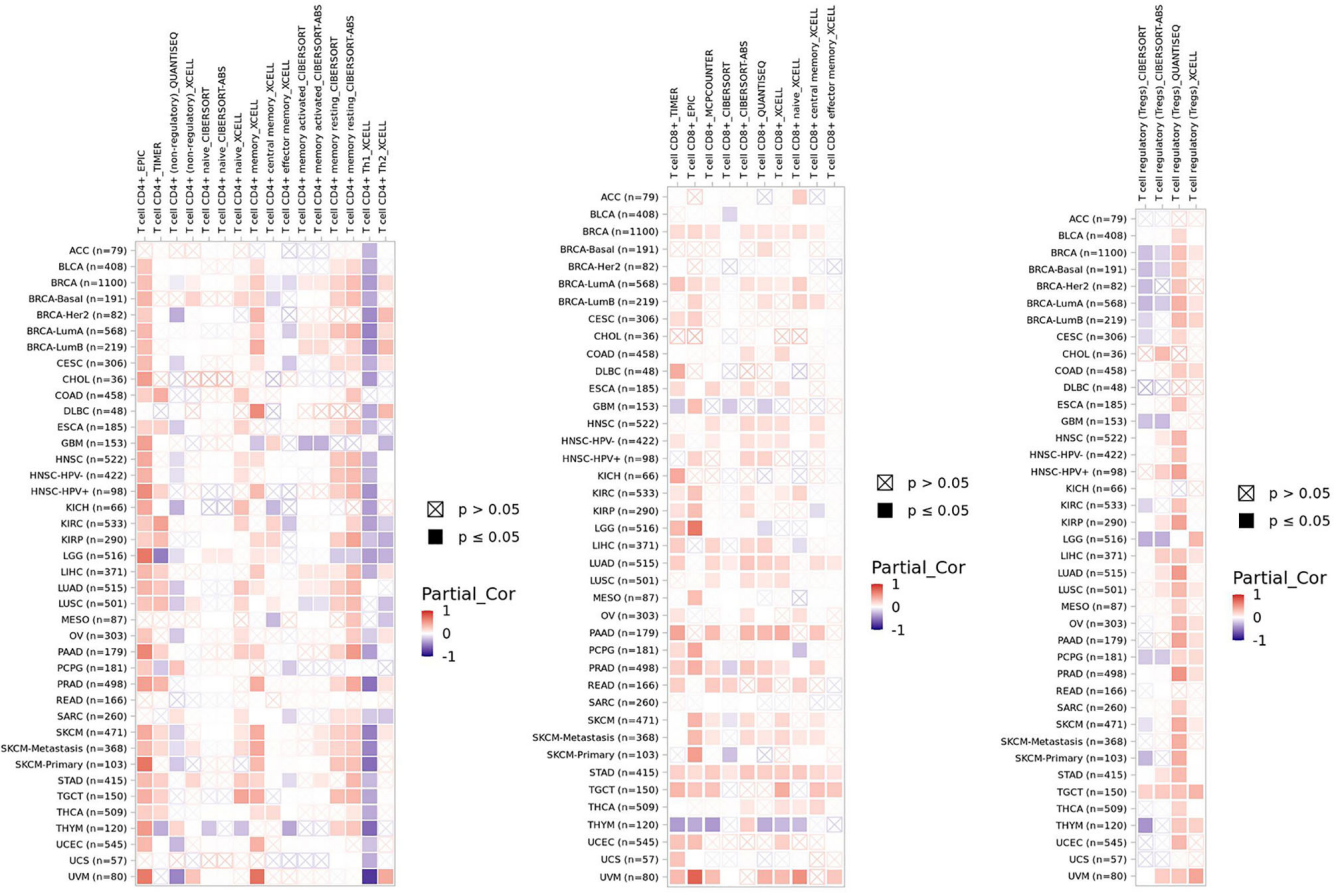
The correlation of FBXW7 expression with CD4^+^ T cells, CD8^+^ T cells, and Tregs infiltration level in diverse cancer types (data from timer 2.0).

FBXW7 deletion in T cells results in enhanced cell proliferation, which expresses both CD4 and CD8 (double-positive, DP); however, the anticipation improvement of single-positive (SP) T cells does not appear as expected, instead DP cells may develop into lymphoma due to uncontrollable cell cycle caused by accumulated c-myc at last (158). It demonstrates that either DP cells are uncapable of performing positive selection or the proliferative and survival ability of SP T cell is injured due to the loss of FBXW7. The former explanation was excluded because positive selection does not take part in cell circle progression, and the latter explanation was remained for it is consistent with the result of experiment. GATA3, a T-cell differentiation regulator, was revealed to be a substrate of FBXW7 taking an effect on the development and differentiation of T cells at the DN (CD4/CD8 double-negative) phase (159). Sox12 is able to promote the degradation of GATA3 mediated by FBXW7 in Th2 cells to inhibit the differentiation of Th2 cells and subsequently weaken allergic inflammation (160). The loss of FBXW7 was also reported to lead to T-ALL (161). NOTCH1/FBXW7 mutation shows a positive correlation with prednisone response against T-ALL (162, 163). The reason patients with FBXW7 mutations demonstrate an incline of prednisone response might lie in the glucocorticoid receptor α (GRα) serving as one of the targets of FBXW7 (164). All mutations of FBXW7 gene are limited within exons 9 and 10, which are the regions functioning to encode the C-terminal binding site to bind substrates (163). Moreover, enhancer of zeste homolog 2 (EZH2) is reported to activate the expression of T-cell multifunctional cytokines and facilitate its survival owing to inhibiting NUMB and FBXW7 that target NOTCH for degradation (165). Here, we show the correlation of FBXW7 expression with CD4^+^ T cells, CD8^+^ T cells, and Tregs infiltration level in multiple cancer types (**Figure 5**).

To summarize, four kinds of immune cells including macrophages, NK cells, B lymphocytes, and T lymphocytes areregulated by FBXW7. Because FBXW7 functions as a regulator of immune cells, we want to figure out how it works in immunotherapy.

### 4.3. FBXW7 Is Involved in Immunotherapy in Multiple Cancers

#### 4.3.1. Renal Cell Cancer

**Figure 6:**
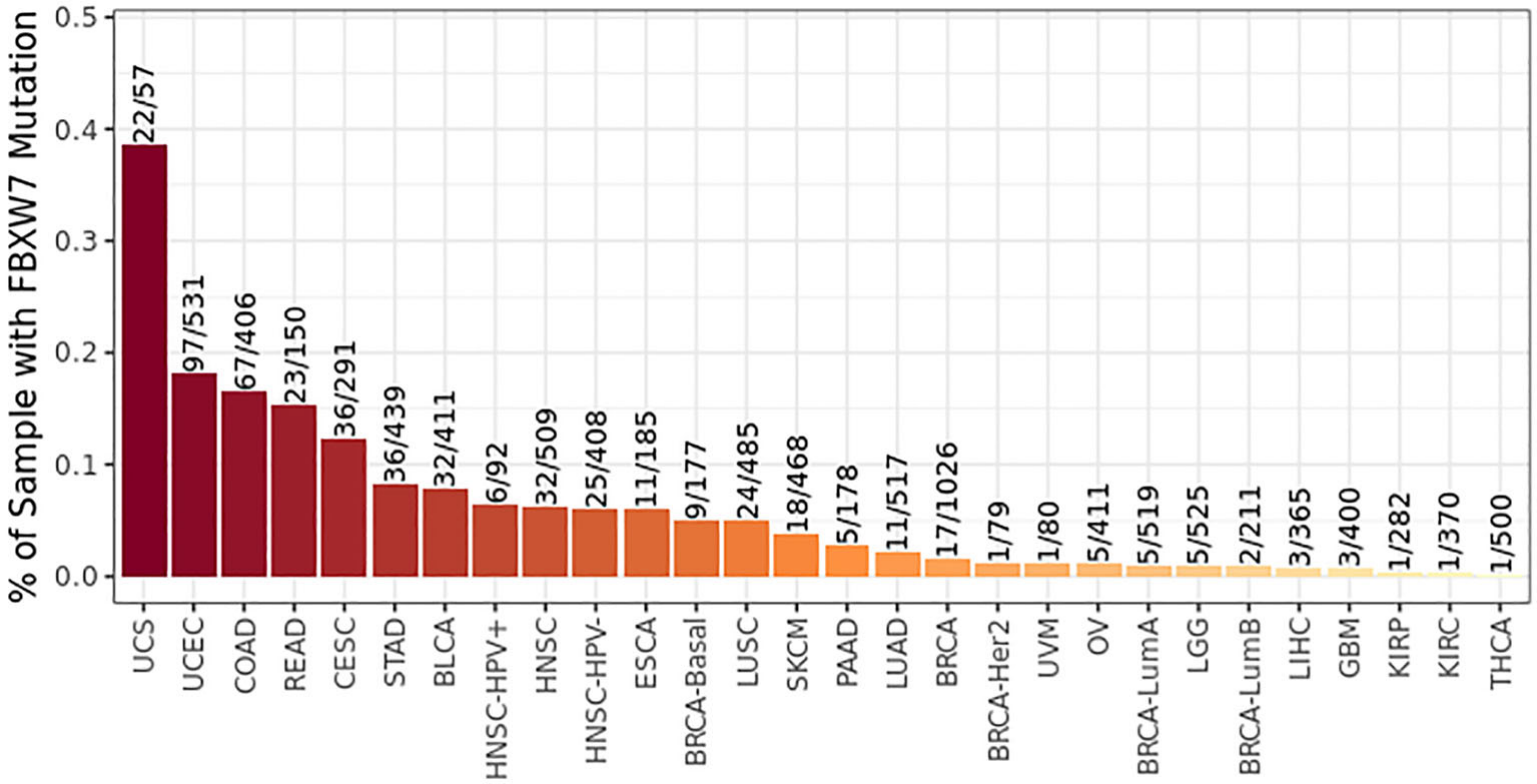
FBXW7 is mutated in multiple cancers (data from timer 2.0).

FBXW7 mutation has been discovered in plentiful human cancers including renal cell cancer (**Figure 6**), and it is one the 10 most frequently mutated genes in metastatic tissues of renal cancer (166). NFAT1 is one of the critical factors of activated T-cell (NFAT) family participating in innate and adaptive immunoreaction (167, 168). NFAT1 improves the expression of PD-L1 by means of boosting TNF abundance in renal cancer to promote the proliferation of renal cancer cells and regulate immunoreaction *via* multiple signaling pathways (169). The expression of PD-L1 regulated by RRM2–ANXA1–AKT axis affects sensitivity to sunitinib and ICIs to cancer cells in renal cell cancer (170). FBXW7 induces the degradation of NFAT1 that is phosphorylated by PI3K/AKT/GSK-3β in a UPS-mediated pathway. Downregulation of the expression of NFAT1 follows with downregulation of PD-L1, which facilitates the tumor cytotoxicity of PD-1 antibodies and infiltration of T lymphocytes (169).

#### 4.3.2 Prostate Cancer

MUC1-C, which could facilitate the expression of IFNGR1 *via* inhibiting FBXW7 expression, drives dedifferentiation of castrate-resistant prostate cancer (CRPC) cell in a chromatin remodeling-dependent way (48). The enhanced stability of IFNGR1 leads to stimulation of the type II interferon-gamma (IFN-γ) inflammatory reaction pathway in prostate carcinomas. In return, the silencing of MUC1-C improves the expression of FBXW7 both at the level of mRNA and proteins. IFN-γ stimulating the IFNGR1 receptor complex induces the transcription factors including Signal Transducer and Activator of Transcription 1 (STAT1) and Interferon Regulatory Factor 1 (IRF1) to actuate the type II IFN response genes and chronic inflammatory reaction in prostate carcinoma cells (48).

#### 4.3.3 Glioblastoma

As a specific inhibitor for NEDD8-activating enzyme, Pevonedistat (also known as MLN4924) is capable of suppressing the degradation function of FBXW7 E3 ligase and leading to the improved stability of c-myc, a critical transcriptional activation factor of PD-L1 binding to the promoter region of PD-L1 gene (171), to promote the expression of PD-L1 (172). In this way, the killing effect of T cells was weakened *via* PD-L1 improvement. However, the inactivation of the cytotoxic effect of T cells induced by Pevonedistat was rescued by PD-L1 blockade. Then, an interesting phenomenon occurs; the treatment of Glioblastoma acquired a better effect through the integration of pevonedistat and anti–PD-L1 drugs than that of each drug alone. In short, the integration of pevonedistat and anti–PD-L1 drugs offers a novel method to cure glioblastoma.

#### 4.3.4 Breast Cancer

HSF1 phosphorylated by gsk3β and ERK1 at Ser303 and Ser307 is revealed as one of the substrates of FBXW7 (173). Nevertheless, the phosphorylation at another amino acid residue, Thr^120^, by PIM2 protects it from being degrading by FBXW7 and results in the accumulation of HSF1, which subsequently induces the expression of PD-L1 in breast cancer and enhances growth of breast cancer (174). This may offer a potential target for anti–PD-L1 drugs treatment.

In addition, the investigation by Singh et al. illustrates that E74-like transcription factor 5 (Elf5) facilitates the expression of FBXW7 through binding to the enhancer region of FBXW7 (51). The deficiency of Elf5 downregulates the expression of FBXW7 and confers the accumulation of IFNGR1 as a result of the deleted ubiquitination of IFNGR1 in breast cancer cells. The IFN-γ signaling pathway therewith is promoted *via* the elevation of IFNGR1 abundance, which facilitates the propagation of neutrophils as well as the potential proliferation and metastasis of breast cancer. The abundance of PD-L1 is improved due to elevation of IFNGR1. As anticipated, the carcinogenicity induced by IFNGR1 could be blocked by PD1 and PD-L1 inhibitors.

#### 4.3.5 Colorectal Cancer

Phoaphoinositide 3-kinase γ (PI3Kγ) is an isotype of PI3K that elicits an effect on the regulation of metabolic pathways in inflammation and oncogenicity (175). PI3Kγ expresses lavishly in macrophages but has no expression in cancer cells (176). As discussed above, an axis concerning FBXW7–MCL-1 is associated with the features of macrophages in colorectal cancer, and suppression of PI3Kγ elicits the reversion of cancer progression in a FBXW7–MCL-1–dependent way (8). PI3Kγ alters the function of macrophages between the status of immunological tolerance and immune surveillance by affecting abundance of cytokines (pro-inflammatory factors: IL-1α, IL-1β, CXCL10, IL-8, and IL-12β; anti-inflammatory factors: TGF-β and IL-10) (8). Hence, PI3Kγ of macrophage is likely to fulfill a function for immunotherapy in colon cancer.

#### 4.3.6 Melanoma

FBXW7 mutation functions as a driver to initiate melanoma by activating NOTCH1 (177). In addition, EZH2 improves the abundance of NOTCH *via* suppressing the inhibitors (NUMB and FBXW7) of NOTCH to attenuate the activation of Bcl-2 and to weaken the polyfunctionality and survival of effector T cells (165). Gstalder et al. found that dysfunction of FBXW7 caused by mutation has a correlation with the resistance to pembrolizumab in melanoma patients (178). To uncover the sealed mechanism, they constructed a FBXW7 deficiency melanoma model in mice and obtained similar consequence as patients. Absence of FBXW7 remolds tumor immune microenvironment into an inclination of weaker response to anti-virus and anti-tumors by means of decreasing IFN-γ–related genes, which are with respect to type I interferon stimulation or viral sensing and the amount of multiple immune cells in tumors. In contrast, the presence of FBXW7 maintaining the stability of Rig-I and melanoma differentiation-associated protein 5 (Mda5) facilitates the dsRNA sensing to further enhance interferon pathway and then boost the sensitivity of anti–PD-1 against tumors. Moreover, the viral sensing pathway could be restored by overexpressing mitochondrial antiviral-signaling protein (Mavs) or interferon regulatory factor 1 (Irf1). Nevertheless, the only fly in the ointment is that the mechanism of FBXW7 affecting Rig-1 and Mda5 still needs further exploration.

#### 4.3.7 Lung Cancer

FBXW7 mutation is associated with unfavorable response to patients with Lung Squamous Cell Carcinoma (LUSC) treated with adjuvant therapy (179), which is conversed to its effects in chemotherapy as a tumor suppression gene. Zhong et al. revealed an evident augment of M2-like TAM and aggressive tumor progression *via* inoculating subcutaneously with Lewis lung cancer cells (LLCs) into mice without myeloid FBXW7 (9). The mechanisms that M2-like TAMs facilitate the propagation and metastasis of LLCs may be as follows. FBXW7 induces degradation of c-myc in a UPS-dependent pathway at the post-translational level. Consequently, the deficiency of FBXW7 results in the accumulation of c-myc, which ulteriorly improves the expression of M2-related genes both at the level of mRNA and proteins. Then, the polarization of M2 macrophages occurs and polarized M2 macrophages facilitate the expression of pro-tumor factors to motivate the progression of LLCs. In this way, chances are that novel targets for tumor immunotherapy are found out. In comparison, a clinical research (180) of non–small cell lung cancer unveiled that patients with mutation of FBXW7 profit more from immunotherapy than those of without mutations, which might be on account of improved infiltration level of M1 macrophages and CD8 T cells as well as the enhanced immunogenicity associated with FBXW7 mutation.

#### 4.3.8 Hematological Malignancies

T-cell receptor (TCR) gene therapy serves as an unconventional immunotherapy that isolates TCR genes from antigen-specific T lymphocytes and then transfer TCR gene into T lymphocytes of patients to amplify abundant antigen-specific lymphocytes (181). Then, antigen-specific lymphocytes are adoptively transferred into patients to exert the function of anti-tumor. As mentioned, FBXW7 is often mutated in hematological malignancies. The mutation of FBXW7 is used to isolate CD8 T cells from healthy donor. CD8 T cells specific for an HLA-A*11:01–presented mutant FBXW7(mFBXW7) peptide were triumphantly isolated, which are capable of recognizing targeting cells edited to express mFBXW7. The recurrent mutation of pR465H in FBXW7 was found to encode an HLA-A*11:01–presented neoepitope, which could be applied into the treatment of hematological malignancies *via* TCR gene therapy.

#### 4.3.9 Coronavirus Disease 2019

Coronavirus disease 2019 (COVID-19), which is caused by severe acute respiratory syndrome coronavirus 2 (SARS-CoV-2), is still a severe ongoing contagious disease causing thousands of millions of deaths worldwide. In human lung cells, RIG-I suppresses the replication of SARS-CoV-2 without the participation of type I/III IFN (182). The 3′UTR of viral RNA is recognized by RIG-I *via* its helicase domains rather than the C-terminal domain. FBW7 is capable of maintaining the stability of RIG-I (178). Therefore, it is possible that FBW7 is capable of interfering with the viral RNA synthesis in the early stage of SARS-CoV-2 invasion *via* stabling RIG-I. Moreover, the expression of PD-1and PD-L1 was reported to increase in patients with severe COVID-19 (183). The abundance of PD-1 demonstrates a closed correlation with the severity of the disease (184). It is rational that ICIs could serve as a promising treatment against COVID-19 in this way. However, patients treating with PD-1/PD-L1 blockade show neither advantages nor disadvantages to their recovery (183).

#### 4.3.10 Others

Furthermore, the mutation of FBXW7 is reported to be associated with immunotherapy resistance in endometrial and pancreatic cancer (185) (details are unknown).

All in all, FBXW7 functions discrepantly in immunotherapy of different cancers. However, it fails to work in COVID-19. More work should be done to investigate the function of FBXW7 in various cancers for so many proteins can be ubiquitinated by SCF^FBXW7^.

## 5. Conclusion

**Figure 7:**
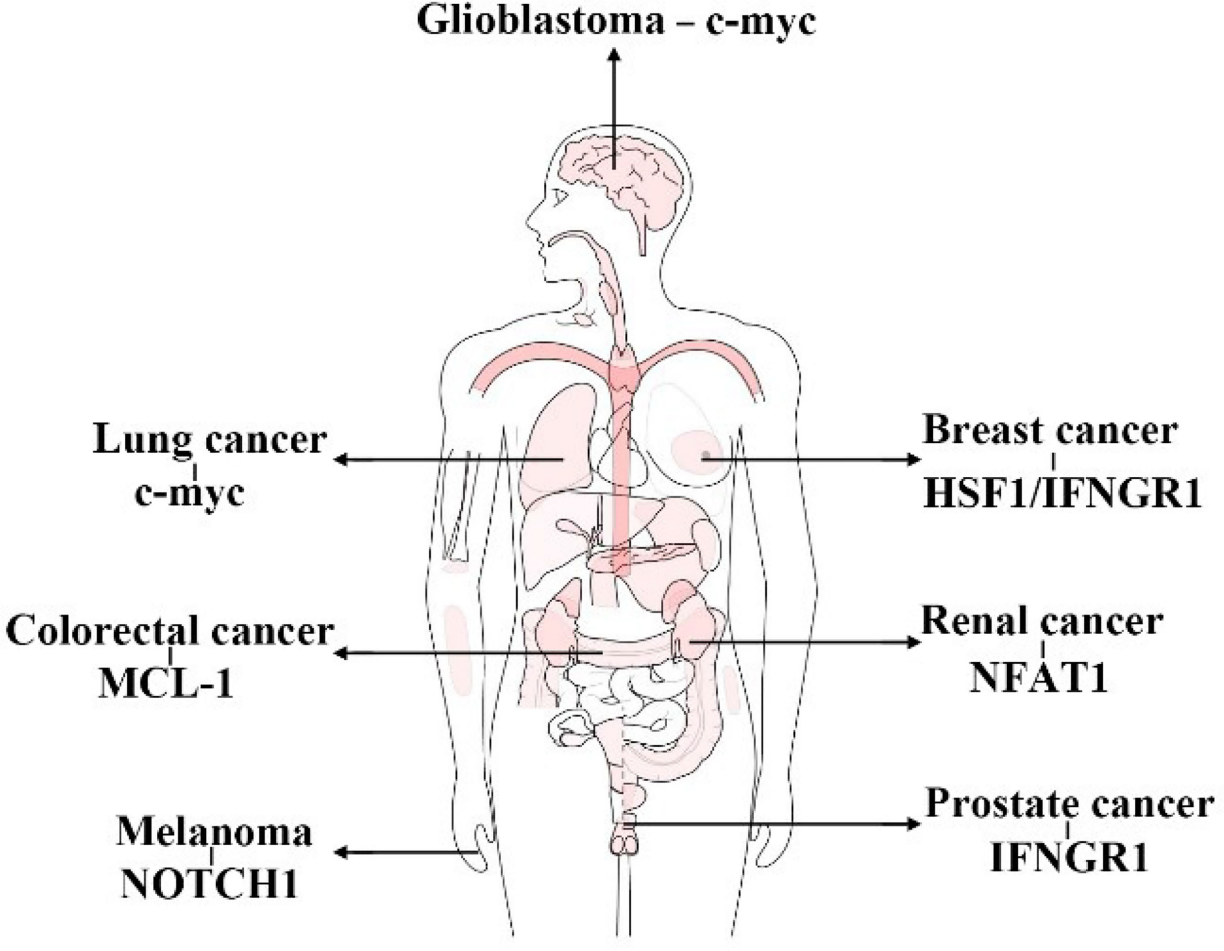
Substrates of FBXW7 functioning in immunity in different cancers. (This figure takes GEPIA as a template).

In general, FBXW7 functions as a suppressor of tumor by means of promoting the degradation of proteins correlated with carcinogenicity, such as c-myc, cyclin E, NOTCH1, and HIF1α (186–191). The mechanism of FBXW7 recognizing proteins and inducing the UPS-dependent degradation has been almost elucidated. Therefore, here, we lay emphasis on its own regulation by various pathways, such as epigenetic regulation, miRNA, circRNA, lncRNA, dimerization, phosphorylation, and autoubiquitination to offer neo-targets for exploring the novel methods of cancer or other diseases treatment *via* regulating the expression of FBXW7. Moreover, previous studies have attached more importance to the effect of FBXW7 on carcinostasis and chemoradiotherapy (64, 117, 192–194) and targeted therapies (195–197), whereas its influence on immunotherapy is ignored to a certain extent. In consequence, we summarize the role of FBXW7 principally in immune cells and in immunotherapy. Here, we demonstrate the relative substrates of FBXW7 functioning in immunity in different cancers (**Figure 7**). In accordance with its role as tumor suppressor, mutation or downregulation of FBXW7 is more likely to contribute to resistance of immunotherapy rather than the opposite. Nevertheless, increasing efforts still need to be taken in unveiling the mysterious regulations, such as how FBXW7 regulating Rig-1 and Mda5 (178). In summary, FBXW7 could be utilized as a target to improve the sensitivity of immunotherapy or that with the combination of other treatment to benefit patients suffering from cancers.

## Author Contributions

JZ and FZ designed this review. YZ and Yinggang Che searched the references. LLX and LX wrote the manuscript. MW and DQ revised the manuscript. YS and HY drew the figures. All authors contributed to the article and approved the submitted version.

## Funding

This work was supported by National Natural Science Foundation of China (81773153).

## Conflict of Interest

The authors declare that the research was conducted in the absence of any commercial or financial relationships that could be construed as a potential conflict of interest.

## Publisher’s Note

All claims expressed in this article are solely those of the authors and do not necessarily represent those of their affiliated organizations, or those of the publisher, the editors and the reviewers. Any product that may be evaluated in this article, or claim that may be made by its manufacturer, is not guaranteed or endorsed by the publisher.
